# Supplementary material for: Evolution of the patella and patelloid in marsupial mammals
Source: PeerJ. 2020 Aug 19;8:e9760. doi: 10.7717/peerj.9760 (PMC7443095; doi:10.7717/peerj.9760)
Supplement: Text S1 — Documentation of museum and literature sources for patellar state scoring; Tables S1–S7: Table S1:Skeletal specimens analysed by micro-computed tomography scans. Table S2: Details of radiographs taken of seven specimens from NHMUK. Table S3: Details of recently deceased (frozen) macropod specimens imaged by medical CT scans. Table S4:Skeletal specimens observed at museum sites. Table S5:UMZC skeletal specimens analysed by micro-CT scans (Table S1). Table S6:Skeletal specimens analysed by x-ray radiography. Table S7:Synthesis of literature on of patellar state (character states and references with notes) for marsupials. [file peerj-08-9760-s001.pdf]

Supplementary Information: Supplementary Text S1- Specimen/Species Information

Alice L. Denyer[1], Sophie Regnault[1,2], John R. Hutchinson[1]

[1] Structure & Motion Laboratory, Department of Comparative Biomedical Sciences, The Royal Veterinary College, Hawkshead Lane, North Mymms, Hatfield, Hertfordshire, AL9 7TA, United Kingdom
[2] Museum of Comparative Zoology and Department of Organismic and Evolutionary Biology, Harvard University, 26 Oxford Street, Cambridge, MA, USA

Corresponding Author:
John R. Hutchinson[1]
Structure & Motion Laboratory, Department of Comparative Biomedical Sciences, The Royal Veterinary College, Hawkshead Lane, North Mymms, Hatfield, Hertfordshire, AL9 7TA, United Kingdom
Email address: jhutchinson@rvc.ac.uk

PeerJ

Supplementary Table S1: Skeletal specimens analysed by micro-computed tomography scans. All specimens were from UMZC, scanned using a Nikon XTek HMX ST 225 scanner on-site, other than the single specimen of *Sarcophilus harrisii*. This specimen was from NMS, scanned at Edinburgh Veterinary School using a Siemens VolumeZoom scanner. One specimen was scanned for each species listed, unless stated otherwise. Specimen numbers in ().

**Genus + species**	**Scan Details**			
	**Voxel size**	**Number of slices**	**Voltage (kV)**	**Current (mA)**
Caenolestes fuliginosus (A8.2/3)	0.0274	1081	100	94
Marmosa carri (A4.33-1)	0.0380	1826	175	170
Marmosa cinera (A4.21-1)	0.0610	1081	110	110
Notoryctes sp. (no number)	0.239	1801	94	238
Dasyurus viverrinus (2 specimens) (A6.11-6; A6.11-4)	0.0980<br>0.0972	1081<br>1081	115<br>115	120<br>120
Myrmecobius fasciatus (A41.2)	0.0547	1081	105	110
Thylacinus cynocephalus (A6.7-1)	0.0685	1081	140	100
Petaurus breviceps (A9.40-2)	0.0478	1081	100	94
Thylogale billardierii (A12.50-3)	0.0963	1081	100	115
Isoodon obesulus (A7.4/5)	0.0759	2000	200	200
Sarcophilus harrisii (no number)	0.391	749	120	185

Supplementary Table S2: Details of radiographs taken of seven specimens from NHMUK. Images were taken using an x-ray unit Solus-Schall Ltd, Type F 1497; and reading unit Dynamix HR² (CR-IR 385). Specimen numbers in ().

**Genus + species**	**Voltage (kV)**	**Current (mA)**	**Exposure Time (seconds)**
Didelphis marsupialis (1959.11.10.1; 1959.11.10.2)	50<br>50	5<br>5	20<br>15
Petaurus sciurus (246A)	50	5	10
Phalanger maculatus (78.2005)	40	5	10
Sarcophilus harrisii (1966.8.11.1; 1968.5.15.2)	50<br>50	5<br>5	20<br>20
Vombatus ursinus (196.4.6.29.1)	50	5	20

Supplementary Table S3: Details of recently deceased (frozen) macropod specimens imaged by medical CT scans. All specimens imaged using 120kV, 100mA and 0.625mm slice thickness. Images taken at the Royal Veterinary College using a GE Medical Systems scanner, LightSpeed RT 16, helical mode, bone reconstruction algorithm. Specimens are numbered "RVC-JRH-Macropus" followed by the specimen number in the table.

**Specimen number**	**Genus + species**	**Number of slices**	**Pixel resolution**
**1**	*Macropus rufogriseus*	592	0.662
**2**	*Macropus rufogriseus*	882	0.676
**3**	*Macropus rufogriseus*	817	1.0469
**4**	*Macropus rufus*– left hindlimb	1476	0.826
**4**	*Macropus rufus*– right hindlimb	1343	0.791

Supplementary Table S4: Skeletal specimens observed at museum sites. Patella state coded 0/1/2 (absent / "patelloid" / ossification present) on the basis of direct observations and, in cases where observations were ambiguous, follow-up CT scanning or radiography. Specimen numbers in ().

<u>NHMUK</u>

**Clade**	**Genus + species**	**Number of specimens**	**Observed patellar state (0/1/2)**
Acrobatidae	*Distoechurus pennatus* (94.2.14.1)	1	1

Dasyuridae	*Dasyurus hallucatus* (19.66.4.42)	1	1
	Dasycercus cristicauda (75.1818)	1	1
	Sarcophilus harrisii (1966.11.1 1968.5.15.2)	2	1
Didelphidae	*Didelphis marsupialis* (93.1.31.1, 1995.63, 1949.1.18.2, 1959.11.10.1, 1959.11.10.2)	5	1
	Didelphis crassicaudata (844.D)	1	1
	Chironectes minimus (849.D)	1	1
	Marmosa elegans (77.7.3.3)	1	1
	Metachirus opossum (65.5.24.5)	1	1
	Caluromys philander (75.1819)	1	1
Macropodidae	*Wallabia bicolor* (1981.958; 1981.955)	2	1
	Macropus agilis (1966.1.7.1; 70.368; 70.367; 1970.2190)	4	1
	Macropus rufus (62.12.26.3; 60.11.4.2; 4.7.12.1; 1961.12.11.2)	4	1
	Petrogale concinna (9.10.3.4)	1	1
	Petrogale inornata (9.10.3.3)	1	1

	Petrogale penicillata (65.10.9.14)	1	1
	Macropus sp. (196; 49.6.20.4; no specimen number)	3	1
	Dendrolagus goodfellowi (1966.5.19.1)	1	1
Notoryctidae	*Notoryctes typhlops* (no specimen number)	1	2
Peramelidae	*Isoodon barrowensis* (13.1.28.8)	1	2
	Perameles nasuta (85.1.10.2)	1	2
	Perameles bougainvillei (70.8.30.1)	1	2
	Isoodon obesulus (5.4.24.10)	1	2
	Perameles lagotis (73.6.21.11; 1948.510.6)	2	2
Petauridae	*Dactylopsula trivirgata* (94.2.14.3)	1	1
	Petaurus scuirus (246A; no specimen number)	2	1
	Petaurus australis (60.4.4.19; 51.4.24.9)	2	1
Phalangeridae	*Petauroides volans* (48.9.6.1; 25.8.1.78; 25.8.1.75; 25.8.1.83)	4	1
	Phalanger maculatus (78.2005)	1	1
	Trichosurus vulpecula (89.266; 212; 53.8.17.2)	3	1

Phascolarctidae	*Phascolarctos cinereus*<br>(253C)	1	1
Potoridae	*Bettongia lesueuri*<br>(277P)	1	1
	Aepyprymnus rufescens<br>(52.5.10.3; 1989.272; 51.4.24.7)	3	1
	Potorous tridactylus<br>(51.4.24.2; 38.10.11.32; 281.A)	3	1
Pseudocheridae	*Pseudocheirus cooki*<br>(51.4.24.6)	1	1
	Pseudocheirus occidentalis<br>(99.4.21.1)	1	1
	Pseudocheirus lemuroides<br>(12.5.5.1)	1	1
Thylacinidae	*Thylacinus cynocephalus*<br>(50.11.22.53; 72.666)	2	1
Thylacomyidae	*"Thylacomys leucura" (Macrotis leucura)*<br>(75.1817)	1	2
Vombatidae	*"Phascolomis tasmaniensis" (Vombatus ursinus)* (196.4.6.29.1)	1	1

UMZC

**Family**	**Genus + species**	**Number of specimens**	**Observed patellar state (0/1/2)**
Caenolestidae	*Caenolestes* sp. (A8.2/3)	1	2 (CT scanned)
Phalangeridae	*Trichosurus vulpecula* (A9.16/8; A9.16/7; A9.16/9; A9.16/15; A9.16/5; A9.16/4; A9.16/6)	7	1
Petauridae	*Petaurus breviceps* (A9.40/2)	1	1 (CT scanned)
Peramelemorphia	*Isoodon obesulus* (A7.4/6; A7.4/7; A7.4/5)	3	2 (CT scanned)
	Perameles gunni (A7.17/2; 7.17.3; 7.17/1)	3	2
	“Thylacomys” (Macrotis) lagotis (A7.1/1)	1	2
Dasyuromorphia	*Phascogale flavipes* (A6.26/1; A6.26/4)	2	1
	Phascogale swainsoni (A6.24/1)	1	1
	Phascogale penicillata (A6.30/1)	1	1
	Dasyurus vivirinus (A6.11/6; A6.11/4)	2	1
	Dasyurus maculatus (A6.10/3)	1	1
	Myrmecobius fasciatus (Mounted specimen)	1 (mounted specimen)	1 (CT scanned)
	Thylacinus cynocephalus (A6.7.5; A6.7/3; mounted specimen)	3 (1 mounted specimen)	1 (CT scanned) in 1 specimen;

			1 in 2 specimens
Macropodidae	*Macropus rufus* (A12.21/2; mounted specimen)	2 (1 mounted specimen)	1
	Macropus malabatus (A12.35/1)	1	1
	Thylogale billardieri (A12.50/2; A12.50/3)	2	1 (CT scanned)
	Onchogalea fraenata (A12.59/3; A12.59/2; mounted specimen)	3 (1 mounted specimen)	1
	Macropus giganteus (A12.17/2)	1	1
Potoridae	*Bettongia lesueuri* (A12.82/4; A12.82/5; A12.82/3)	3	1
	Aepyrymnus rufescens (A12.77/1; A12.77/3; A12.77/4)	3	1

OUMNH

**Clade**	**Genus + species**	**Number of specimens**	**Observed patellar state (0/1/2)**
Didelphidae	*Caluromys lanatus* (8155)	1	1
Macropodidae	*Macropus cangaru* (9061)	1	0/1
	Macropus rufogriseus (9379; 8037; 2887; 8039; 8069; 8068)	6	1
Thylacomyidae	*Macrotis lagotis* (4820)	1	2

Phalangeridae	*Trichosurus vulpecula*<br>(4854; 4047)	2	1

<u>NMS</u>

**Clade**	**Genus + species**	**Number of specimens**	**Observed patellar state (0/1/2)**
Dasyuridae	*Sarcophilus harrisii*<br>(Ex 2.9)	1 (mounted specimen)	0/1
	Dasyurus maculatus<br>(XH2-10.6)	1	0/1
Didelphidae	*"Didelphis"*<br>(Ed. U. Anat. C9)	1	0/1
	Didelphis marsupialis<br>(1879-8-70)	1	1
Macropodidae	"Kangaroo", sp. Unknown<br>*(B4)*	1	1
	Macropus rufogriseus<br>(mounted specimen)	1 (mounted specimen)	0/1
	Setonix brachyurus<br>(Z2013.120.1)	1	0/1
	"Grey kangaroo" *(Macropus giganteus* or *fuliginosus)*<br>(Ex 2.9 Display 2008)	1	0/1
	Dendrolagus ursinus<br>(1881-3-1)	1	1
Notoryctidae	*Notoryctes* sp.<br>(Ex G9)	1 (mounted specimen)	2
Phalangeridae	*Trichosurus vulpecula*<br>(Z 1905.063)	1 (mounted specimen)	1
	Phalanger sp.<br>(1931-120-41)	1	0/1

Thylacinidae	*Thylacinus cynocephalus*<br>(1863-30-2)	1	0/1
Vombatidae	*Lasiorhinus latifrons*<br>(1868-31)	1	0/1

Supplementary Table S5: UMZC skeletal specimens analysed by micro-CT scans (Table S1). Patellar state was scored, on the basis of scan findings, as 0/1/2 (absent / "patelloid" / ossification present). Specimen numbers in ().

**Genus + species**	**Observed patellar state (0/1/2)**
Caenolestes fuliginosus (A8.2/3)	2
Marmosa carri<br>(A4.33-1)	1
Marmosa cinera<br>(A4.21-1)	1
Notoryctes sp.<br>(no number)	2
Dasyurus viverrinus (2 specimens)<br>(A6.11-6; A6.11-4)	1
Myrmecobius fasciatus<br>(A41.2)	1
Thylacinus cynocephalus<br>(A6.7-1)	1
Petaurus breviceps<br>(A9.40-2)	1
Thylogale billardierii<br>(A12.50-3)	1
Sarcophilus harrisii<br>(no number)	1
Isoodon obesulus (A7.4/5)	2

Supplementary Table S6: Skeletal specimens analysed by x-ray radiography. Presence or absence of ossified patellar tissue was determined from each image. NHMUK specimen numbers in ().

**Genus + species**		**Number of specimens**	**Ossified tissue (Present/Absent)**
Petaurus scuirus (246A)		1	Absent
Phalanger maculatus (78.2005)		1	Absent
Vombatus ursinus (196.4.6.29.1)		1	Absent
Sarcophilus harrisii (1966.8.11.1; 1968.5.15.2)		2	Absent
Didelphis marsupialis (1959.11.10.1; 1959.11.10.2)		2	Absent

Supplementary Table S7: Synthesis of literature on of patellar state (character states and references with notes) for marsupials. Codings for stem Metatheria followed Samuels et al. (2017); i.e. ossified patella in sparassodonts such as *Arctodictis*, *Callistoe*, *Prothylacinus* and *Sipalocyon*; perhaps also *Mayulestes* (e.g. Sinclair, 1905; Wood, 1924; Argot, 2004—but see other character state codings in Forasieppi, 2009); but unclear (possibly patelloid) in others such as *Borhyaena*, *Cladosictis* and *Lycopsis* (Argot, 2004). See main text for references.

**Clade**	**Genus + species**	**Patellar state 0/1/2**	**References**	**Nature of specimens/Comments**
**Caenolestidae**	*Rhyncholestes raphanurus*	2	Szalay & Sargis (2001)	Museum and fresh skeletal specimens of a range of species examined; unknown age/quality/quantity. Patella described as present/absent with no reference to fibrocartilage patelloid.
	Caenolestes (presumed *C. fuliginosus*)	2	Szalay & Sargis (2001)	[As above.]
	Caenolestes (presumed *C. fuliginosus*)	2	Osgood (1921)	"Two entire specimens representing both sexes and two others with some parts mutilated were preserved in formaldehyde and bichloride of mercury. In addition, several dry skeletons and a small number of conventional skins and skulls were saved."
**Didelphidae**	38 Didelphidae spp. across 18 genera	0	Flores (2009); also Forasieppi (2009)	Postcranial characters of morphology examined in (skeletal?) museum specimens of 38 Didelphidae spp. across 18 genera. Several hindlimb characters examined in a number of these specimens. Only note on patella: "the patella is absent in didelphids".
	Didelphis virginiana	0/2? Nodules of ossification in some (coded here as 1/2)	Szalay & Sargis (2001)	Visually examined ontogenetic series of skeletal specimens of 5 ages (uncertain quantity and quality of specimens).<br>"It should be noted that in occasional specimens of *D. virginiana* (probably old individuals in a species with a short lifespan) an elongated curved ossification inside the tendon can be seen."
		1	Wood (1924)	"Unossified"
		1/2?	Diogo et al. (2016)	Dissection of 5 adult *D. virginiana*. Study of myology. "[the 3 muscle bodies of QF] are blended distally and insert together on the patella and tibia"

	Didelphis albiventris	1	Inamassu et al. (2017)	16 adults; histology, radiography and CT. No indication of ossification. CT showed area of greater soft tissue density in region of true patella. Centre of tendon contained “classic fibrocartilage pad”.
	Philander sp.	1	Wood (1924)	“Unossified”
	Marmosa chapmani	1	Wood (1924)	“Unossified”
	Metachirus nudicaudatus	0	Szalay & Sargis (2001)	[As above.]
**Notoryctidae**	*Notoryctes typhlops*	2	Johnson & Walter (1989)	Based on early anatomical studies (Thomas, 1920; Osgood, 1921) and specimens examined.
		2	Thompson & Hillier (1905)	Dissection of 3 specimens; primarily examination of the myology of the hindlimb. Multiple references to insertion of muscles on the patella.
	Notoryctes spp.	2	Warburton (2006)	Extensive anatomical descriptions based on CT, radiography and dissection of specimens of both *Notoryctes* spp.
**Peramelidae**	*Isoodon obesulus*	2	Reese et al. (2001)	1 juvenile female; histological studies.
	Perameles nasuta	2	Reese et al. (2001)	1 adult male; histological studies.
		2	Vogelnest & Allan (2015)	Radiographic study; single specimen. Strongly reinforced by older osteological literature such as Osgood (1921).
**Thylacomyidae**	*Macrotis lagotis*	2	Vogelnest & Allan (2015)	Radiographic study; single specimen.
**Thylacinidae**	*Thylacinus cynocephalus*	1	Cunningham (1882); also supported by Wood (1924) and Warburton et al. (2019)	p.38: “In neither animal is there an osseous patella, but the different portions of the quadriceps, together with the sartorius, unite to form an exceedingly dense and thick tendinous expansion in front of the knee-joint. This is so tough and resistant to the knife that it almost resembles fibro-cartilage in its consistence.”
**Dasyuridae**	*Dasycercus byrnei*	1	Reese et al. (2001)	1 adult (4 years) male; histological studies.
	Dasyurus viverrinus	1	Reese et al. (2001); also Wood (1924)	1 adult female; histological studies.
	Dasyurus sp.	1/2	Forasieppi (2009)	data matrix coding (most other crown Marsupialia = 0/1; not 2)

	Dasyurus maculatus	0/1	Vogelnest & Allan (2015)	Radiographic study; single specimen.
	Phascogale tapoatafa	1	Reese et al. (2001); also Wood (1924)	1 adult male; histological studies.
	Antechinus swainsonii	1	Reese et al. (2001); also Wood (1924) for *A. laniger*	1 female (1 year old); histological studies.
	Sminthopsis crassicaudata	1	Wood (1924)	"Unossified"
	Sarcophilus harrisii	1	Wood (1924)	"Unossified"
		0/1	Vogelnest & Allan (2015)	Radiographic study; single specimen.
**Microbiotheriidae**	*Dromiciops gliroides*	0	Szalay & Sargis (2001)	[As above.]
**Phascolarctidae**	*Phascolarctus cinereus*	1	Reese et al. (2001)	1 juvenile male; histological studies.
		0	Lee & Carrick (1989)	(with ref to Sonntag 1922) "the most distinctive features to emerge from this study are that no patellae, even a cartilaginous one, is present"
		0	Waterhouse (1846)	"[the koala and wombat] agree in the non-possession of a patella" (examination of museum specimens from Australia)
		0/1	Vogelnest & Allan (2015)	Radiographic study; single specimen.
**Vombatidae**	*Lasiorhinus latifrons*	1	Reese et al. (2001)	1 adult female; histological studies.
		0/1	Vogelnest & Allan (2015)	Radiographic study; single specimen.
	Vombatus ursinus	0/1	Vogelnest & Allan (2015)	Radiographic study; single specimen.
		0/1	Home (1808)	p.306: "There is no patella; but the tendon of the extensor muscles of the leg, where that bone is usually situated, is much thickened."
		0	Waterhouse (1846)	"The patella, or knee bone, is wanting"<br>Examination of museum specimens from Australia.
		1	Macalister (1870)	p.169: "The patella is mentioned as absent in the Wombat by Sir E. Home (Phil. Trans. vol. xcviii. 1808, p. 304); in reality it is present, but cartilaginous."
**Burramyidae**	*Burramys parvus*	1	Reese et al. (2001)	1 x adult male; histological studies.
**Phalangeridae**	*Trichosurus vulpecula*	1	Reese et al. (2001)	2 x juvenile + 4 x adult + 1 unknown age; histological studies.

	Spilocuscus maculatus	1	Cunningham (1882)	p.38: "In neither animal is there an osseous patella, but the different portions of the quadriceps, together with the sartorius, unite to form an exceedingly dense and thick tendinous expansion in front of the knee-joint. This is so tough and resistant to the knife that it almost resembles fibro-cartilage in its consistence."
**Acrobatidae**	*Acrobates pygmaeus*	1	Reese et al. (2001)	1 adult female; histological studies. (classified as Burramyidae in this paper)
**Tarsipedidae**	*Tarsipes rostratus*	2 (coded here as 1/2)	Waterhouse (1846)	"The patella is present" Examination of one female specimen preserved in spirit.
	Tarsipes sp.	2 (coded here as 1/2)	Osgood (1921)	p. 94: " among living marsupials the only others having an osseous patella are *Tarsipes*..."
**Petauridae**	*Petaurus norfolcensis*	0/1	Vogelnest & Allan (2015)	Radiographic study.
**Pseudocheridae**	*Pseudocherius peregrinus*	1	Reese et al. (2001)	1 x juvenile male; histological studies.
		0/1	Vogelnest & Allan (2015)	Radiographic study.
**Hypsiprymnodontidae**	*Hypsiprymnodon moschatus*	1	Reese et al. (2001)	1 x adult male; histological studies.
**Potoroidae**	*Bettongia* sp.	1	Reese et. al (2001)	3 x adults; histological studies.
	Aepyprymnus rufescens	1	Reese et al. (2001)	1 x adult male; histological studies.
**Macropodidae**	*Macropus rufus*	1	Holladay et al. (1990)	3 *Macropus* spp. Unknown ages.<br>Radiographic study: no ossification. Histological study: presence of fibrocartilage pad.
		1	Reese et al. (2001)	1 x 3 year female; radiographic study.<br>2 x juvenile + 4 x adult; histological studies.
		1/2?	Windle & Parsons (1898)	Dissection of one juvenile (from pouch) and one adult immature animal. References to insertion of tendons/origins of muscles on/from the "patella" (no description of whether osseous).
		0/1	Vogelnest & Allan (2015)	Radiographic study.
	Macropus rufogriseus	1	Holladay et al. (1990)	3 *Macropus* spp. Unknown ages.<br>Radiographic study: no ossification. Histological study: presence of fibrocartilage pad.
		1	Reese et al. (2001)	1 x juvenile male; radiographic study.<br>1 x juvenile + 1 adult female; histological studies.

	Macropus eugenii	1	Reese et al. (2001)	1 adult male; radiographic study.<br>2 x adult female; histological studies.
	Macropus giganteus	1	Reese et al. (2001)	1 juvenile male; radiographic study.<br>1 x juvenile + 3 x adults; histological studies.
	Macropus robustus	1	Reese et al. (2001)	2 adult males; histological studies.
	Macropus fuliginosus	1	Reese et al. (2001)	4 x adults; histological studies.
	Macropus dorsalis	1	Reese et al. (2001)	1 adult male; histological studies.
	Dendrolagus dorianus	1	Reese et al. (2001)	1 x juvenile + 2 x adults; radiographic study.<br>1 adult female; histological studies.
	Dorcopsis muelleri /veterum	1	Reese et al. (2001)	3 x juvenile + 2 x adults; radiographic study.
	Dendrolagus goodfellowi	0/1	Vogelnest & Allan (2015)	Radiographic study; single specimen.
	Thylogale billardierii	1	Reese et al. (2001)	1 juvenile male; radiographic study.
	Thylogale stigmatica	1	Reese et al. (2001)	1 adult male; histological studies.
	Petrogale persephone	1	Reese et al. (2001)	1 adult male; histological studies.
	Petrogale concinna	1	Reese et al. (2001)	1 female unknown age; histological studies.
	Onchogalea sp.	1	Reese et al. (2001)	1 adult male; histological studies.
	Setonix brachyurus	2 (coded here as 1/2)	Dawson et al. (1989)	“[muscles of the QF group] insert via a common tendon, which encloses the patella, into the upper end of the tibia.”<br>“the patella is an ossification in the tendons of the muscles which pass over the knee joint” (diagram of Quokka including patella)
	Setonix brachyurus	0/1	Vogelnest & Allan (2015)	Radiographic study; single specimen.
	Wallabia bicolor	0/1	Vogelnest & Allan (2015)	Radiographic study; single specimen.
