# Supplementary figures and images for: Evolution of the patella and patelloid in marsupial mammals
Source: PeerJ. 2020 Aug 19;8:e9760. doi: 10.7717/peerj.9760 (PMC7443095; doi:10.7717/peerj.9760)

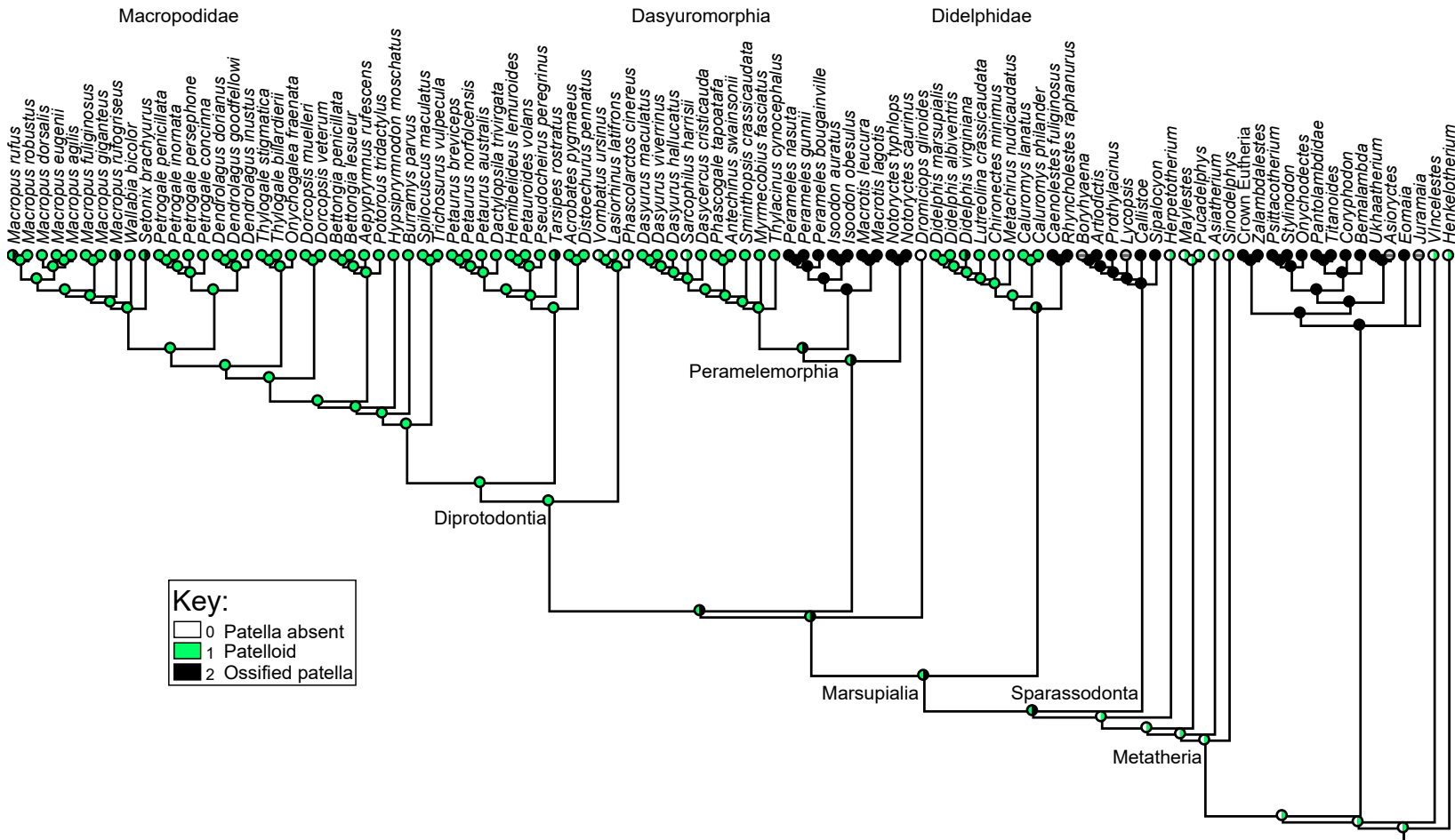

Supplement: Figure S1 — Using the fossil outgroups adopted by Samuels, Regnault & Hutchinson (2017), unordered maximum parsimony algorithm, and rescoring all state “0” fossil taxa as “0/1” (see Methods). See Results for interpretations. [file peerj-08-9760-s003.pdf]
